# Epidemic Cholera: Its Pathology and Treatment

**Published:** 1866-08

**Authors:** R. Dexter

**Affiliations:** Chicago, Ill.


*Epidemic Cholera: Its Pathology and Treatment.* By R. DEXTER, M. D., of Chicago, Ill.

If we carefully trace the gradual digression from a truly physiological action through the entire changes of its pathological course, in a typical case of cholera, we shall find about the following order of events:

1. Slight derangement of the alimentary canal, muscular languor and tremulousness, restlessness and headache.

2. An enhanced condition of the preceding phenomena, with abdominal uneasiness, perhaps actual vomiting and purging, tongue coated, cramping in distal and peripheral parts, arrest of secretion of urine, with coldness of surface.

3. Active vomiting, purging of the characteristic rice-water dejection, respiration labored and abridged, excessive and painful cramping of both body and extremities, great coldness of surface, and the pulse variable.

4. Dyspnœa very great, peripheral circulation nearly suspended, venous congestion, labored cardiac action, skin livid, cold and bathed in perspiration, great precordial pain and coldness of respired air.

5. Low, irritative fever, with an irritated and congested condition of one or more vital organs.

Many prominent men claim that the specific action of the poison is upon the capillary circulation, some say pulmonary, others general; to this I must in part disagree. That the capillary system is affected, is unquestionably correct; but it seems to me that there is as much evidence to confirm an opinion that its primary action is upon the nervous, glandular or cellular systems, or that it acts through the blood upon any other system, as upon capillaries alone. Now my belief is, that as soon as any poison has entered the blood, it acts upon its composition and changes its constituent elements; that it changes the constituent elements of the cells and their contents; that this modifies *absorption* and *secretion*, and thus alters *assimilation* and *excretion*. I also believe that each known hæmatic poison has not only some individual peculiarities but many properties in common with other poisons; and that any morbific agent taken into the blood must be eliminated by some of the emunctories. Some of these have a specific tendency to escape through one eliminating channel rather than through others; and that when one or more of the excretory organs are dormant, a compensating action is established, by which the others eliminate whatever the disabled organs fail to remove.

These principles are as well illustrated and as well settled, in respect of the elimination of poisons, as they are in the more ordinary every-day work of excretion.

I will here cite the action of antimony, and compare it with that of the cholera poison, assuming that they are both taken into the blood slowly, so that the antimony is received into the blood about as cholera poison usually is. Their similar actions are prostration, irritation of the alimentary canal, impaired function of the pneumogastric nerve and other organic nerves, impoverishment of the blood, lowering of animal heat, excessive vomiting and purging, and cold, clammy perspiration.

Bichloride of mercury, in medicinal doses, acts as a laxative, cathartic, eccritic and catalytic hæmatic. It is eliminated by the bowels, liver, kidneys, salivary glands, etc. In poisonous doses, it is a violent irritant and caustic, creating vomiting and purging by its caustic properties.

That there is in cholera an early arrest of functional and capillary action, and consequent elimination, is proved by the following conditions:

1. An arrest of secretion, viz., urine and bile.
2. A gradual subsidence of animal heat.
3. An imperfect æration of blood.
4. Undue fulness of larger vessels.

For a further confirmation of the preceding principles, the reader is referred to Dr. F. W. Headland's work on the action of medicines. Judging from the above facts, it seems to me that the indications for treatment are as patent as are the principles of medicine. They are to restore the action of the emunctories, by recalling to the surface the receding capillary circulation. This is to be done by allaying nervous irritation, by artificially restoring animal heat, and by the administration of eliminating medicines. Nervous irritation is probably best allayed by opium, in regular and sustained stimulant doses, until capillary action is provoked. Perhaps the preferable preparations are the tincture and camph. tincture of opium, (if the discharges are excessive,) with acetate of lead and acetate of morphine injections. Probably the best way to restore the animal heat is to completely envelope the patient (except his face) in wet blankets, as hot as can be tolerated.

This combined action will generally suffice to restore normal circulation and elimination; if not, we can resort to the use of the true eliminating medicines.

My selection is usually a mercurial, because it, *per se*, has the very effect we should produce,—cathartic, catalytic and eliminative. But no definite rule can be established as to medicines, as each prescription must be modified by the present and previous conditions of the patient.

Here, perhaps, I should state, that I administer the cathartic as soon as excessive action is checked, and give such other eliminatives as I deem the case requires.

But if the disease has advanced to desperate vomiting, purging and cramping, we should employ our revulsive measures with great activity, allowing ice and cold water internally, with sinapisms to the epigastrium and precordia.

But in a state of collapse, where the watery portion of the blood has been drained away by the excessive evacuations, and where prostration marks every organ of the body, it is my opinion that little can be done except by supplying such nourishment as requires but little digestion, and will serve to sustain vital and recuperative action,—beef essence, fresh milk, coffee or tea, and chicken broth, etc. Chloroform, I think, is contraindicated by a highly-carbonized condition of the blood, by an already embarrassed condition of the organic nerves, and by an oppressed condition of the heart and lungs.

Perhaps I ought here to mention some of my reasons for considering the rice-water dejections the result of pressure. The abdominal veins are congested, and with such congestion we always have an aqueous exudation. Anatomically, this is the most copiously exuding surface in the body. Hydrogogue cathartics are probably exuded into the lower portion of the intestines as a result of congestion. Nervous shocks, as fear, cold and the like, produce such action, especially when the kidneys are inactive, as seems to be the case in cholera.

